# Abnormal Locations of Thymic Tissue as an Uncommon Cause of Neck Masses in Children: A Practical Approach

**DOI:** 10.3390/life13030685

**Published:** 2023-03-02

**Authors:** Laura Andreozzi, Chiara Sacchi, Carlotta Biagi, Arianna Dondi, Michelangelo Baldazzi, Filomena Carfagnini, Laura Greco, Donatella Vivacqua, Marcello Lanari

**Affiliations:** 1 Pediatric Emergency Unit, IRCCS Azienda Ospedaliero-Universitaria di Bologna, 40138 Bologna, Italy; 2 Specialty School of Paediatrics—Alma Mater Studiorum, University of Bologna, 40126 Bologna, Italy; 3 Pediatric and Adult Cardio Thoracic and Vascular, Oncohematologic and Emergency Radiology Unit, IRCCS Azienda Ospedaliero Universitaria di Bologna, 40138 Bologna, Italy

**Keywords:** cervical masses, ectopic thymus, thymus herniation, ultrasound, children

## Abstract

Background: The thymus gland is a lymphoid organ normally located in the anterior mediastinum. Location abnormalities of the thymus, such as ectopic thymus or the superior herniation of a mediastinal thymus, could be responsible for the occurrence of cervical masses in pediatric patients, raising concerns among clinicians. The knowledge of these conditions is essential for a thorough differential diagnosis and for preventing unnecessary invasive procedures. Methods: Descriptive retrospective series of three patients with a cervical mass, that was later diagnosed as ectopic thymic tissue. Results: The thymus has a unique and distinctive ultrasound appearance that is the keystone to detecting thymic parenchyma in locations other than anterior mediastinum. In selected patients, an accurate ultrasound could be conclusive for the diagnosis, with no need for further and potentially risky procedures. Conclusions: This case series supports the use of ultrasound in both diagnosis and follow-up of thymus location abnormalities, advocating a minimal invasive and conservative approach.

## 1. Introduction

The thymus gland is a lymphoid organ usually located in the anterior mediastinum. It is a paired organ that is recognizable early in the 6th week of fetal life. The thymus originates embryologically from the ventral wings of the third pharyngeal pouch on each side. During the 8th week, each thymic primordium elongates caudally and medially and forms the thymopharyngeal duct. As the primordial thymus descends into its final position within the anterior superior mediastinum, the upper extremity of the thymus is drawn out and eventually disappears [1]. The thymus is an important immune organ during childhood. As a primary lymphoid organ, its principal role is the proliferation and maturation of lymphoid progenitors or thymocytes [2]. Abnormal thymus embryogenesis and migration could be responsible for some thymus congenital abnormalities. Since most of these conditions are not symptomatic, they are probably underdiagnosed, and their incidence is likely underestimated [3].

Among thymus congenital anomalies, abnormal locations, such as ectopic cervical thymus (ECT) or the superior herniation of a normal mediastinal thymus, could be responsible for the occurrence of cervical masses in pediatric patients.

Neck masses are common in pediatric population. Although in most cases they are benign conditions, physicians should be aware that 12% of neoplastic diseases in pediatric age are located in the head and neck [4].

The knowledge of abnormal locations of thymic tissue is essential for a thorough differential diagnosis and for preventing unnecessary invasive procedures.

Ultrasound and magnetic resonance imaging (MRI) are both useful for the diagnosis of abnormal locations of thymic tissue. While ultrasound exams are often rapid, well-tolerated and easy to perform in the majority of children, MRI examinations usually require deep sedation or general anesthesia at this age.

## 2. Methods

We report three pediatric clinical cases of congenital thymus location abnormalities along with their clinical and radiologic features, showing that an accurate ultrasound could have been conclusive for the diagnosis, with no need for further investigations. This case series supports a conservative and minimal invasive approach in both diagnosis, management and follow-up of thymus location abnormalities. The study was performed in accordance with the World Medical Association Declaration of Helsinki, and patient confidentiality was protected. The specific approval of an Ethics Committee was not required for this noninterventional case series based on retrospective, anonymized data; nevertheless, informed consent was obtained from parents for publication.

## 3. Report of Cases

### 3.1. Case 1

A 5-year-old previously healthy girl was admitted to our Pediatric Unit for a recent history of an anterior neck swelling appearing during coughing, that brought family and physician’s concerns. She had no history of fever, dysphagia, wheeze, stridor or other signs of respiratory distress. Her past clinical history was unremarkable. A painless, non-pulsatile, 4 cm × 4 cm swelling, protruding above the suprasternal notch during coughing, was found on clinical examination. The skin above the mass was preserved. No localized or generalized lymphadenopathy was found on physical examination. Blood tests were within normal limits, including complete blood cell count, liver and kidney function, markers of inflammation and the immunological panel. A static ultrasound of the neck was performed, without abnormal findings.

**Figure 1:**
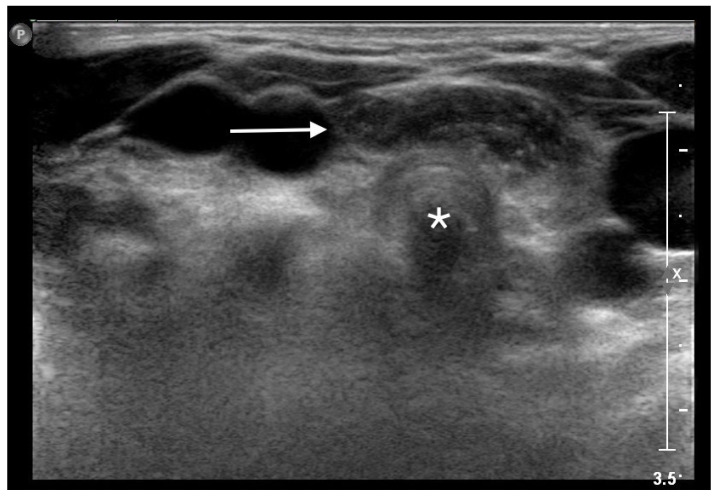
Superior herniation of the normal mediastinal thymus in a 5-year-old girl. Transverse sonogram obtained during the Valsalva maneuver showing a roughly oval structure (*white arrow*) with the typical “starry sky” appearance of thymus, in front of the cervical trachea (*white star*).

An MRI of the neck, carried out under general anesthesia, showed an orthotopic thymus, located into the anterior mediastinum, without any mass effect. After the MRI, a dynamic ultrasonography was carried out during the Valsalva maneuver, showing the superior herniation of a mass with the sonographic appearance of the normal thymus. The mass consisted of a roughly oval structure, with multiple linear hyperechoic septa, and scattered hyperechoic foci, giving a characteristic “speckled” or “starry sky” appearance. The ultrasound examination showed the intermittent superior movement of the thymus into the cervical region, reaching the cervical trachea, without any buckling or displacement of adjacent structures (Figure 1).

At clinical and sonographic 3-year follow-up the size of the swelling was decreased, due to the physiological involution of the thymus.

### 3.2. Case 2

A previously healthy 1-year-old child presented with a 10-day history of a palpable mass on his left side of the neck, with no other signs or symptoms. On clinical examination, the cervical mass appeared painless, tender to the touch, movable in the surrounding tissue. The remainder of the child’s general physical examination was unremarkable. Blood tests, including screening tests for immune function deficiency, were normal.

**Figure 2:**
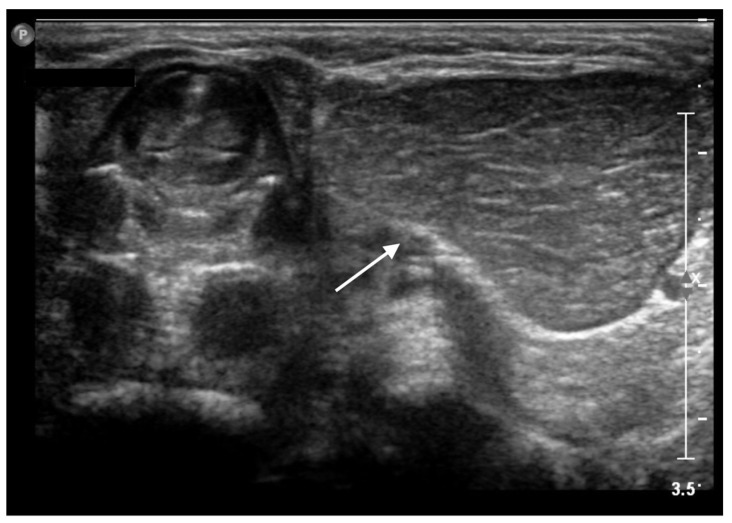
Transverse sonogram showing a 2 × 3 cm hypoechoic mass (*white arrow*) with linear hyperechoic foci, located to the left side of the cervical trachea.

The ultrasound showed a 2 × 3 cm hypoechoic mass with consensual vascular signal, located to the left side of the cervical trachea. It had a relatively homogeneous background echogenicity, with multiple linear hyperechoic septa (Figure 2).

**Figure 3:**
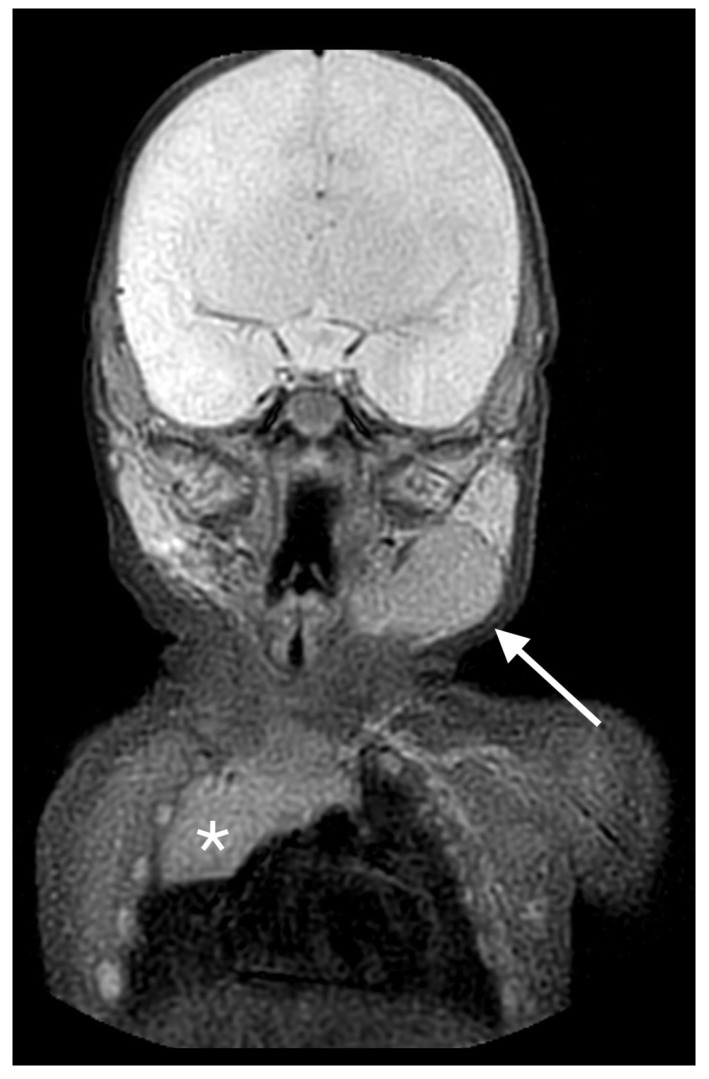
STIR sequences showing a left cervical mass (*white arrow*), isointense to the mediastinal thymus gland (*white star*).

The child was admitted to our Pediatric Unit for further investigation. On clinical examination, the cervical mass appeared painless, tender to the touch, movable in the surrounding tissue. The remainder of the child’s general physical examination was unremarkable. Blood tests were within normal limits in relation to age. An MRI under anesthesia was carried out, showing an oval mass in the left side of the neck, just beneath the submandibular gland, without significant mass effect. The lesion appeared isointense to the thymus gland on all sequences and showed a slight enhancement in the central area, being compatible with ectopic thymus gland. T2-weighted scans showed a well-demarcated quadrilateral-shaped lesion with homogeneous intermediate signal intensity, consistent with ECT in the left latero-cervical region. On Short TI Inversion Recovery (STIR) MRI sequences, the mass appeared isointense to the mediastinal thymus gland (Figure 3).

Given the benignity of this condition and the physiologic involution of the thymus, no further investigation or therapy were needed. An ultrasound and clinical follow-up was started 6 months and 1 year later, respectively, documenting a progressive shrinkage of the lesion.

### 3.3. Case 3

**Figure 4:**
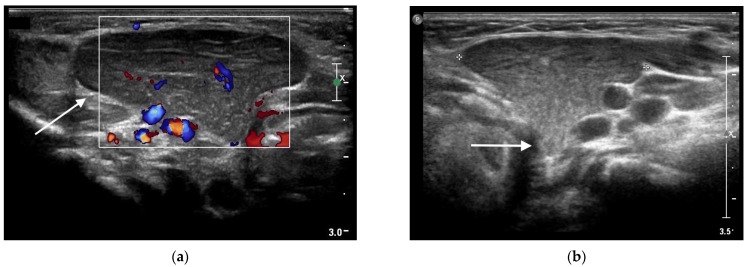
Ectopic cervical thymus in a 11-month-old toddler accidentally noticed on clinical examination for an acute upper respiratory infection. (**a**) Longitudinal sonogram showing a 3.5 × 1.7 cm hypoechoic lesion (*white arrow*), with intralesional vascular signs and punctate and linear echogenic foci resulting in a “speckled” or “salt and pepper” pattern, compatible with ectopic cervical thymus. (**b**) Portion of thymic tissue (*white arrow*) extending into deeper layers, likely connecting the ectopic mass to the orthotopic thymus.

A 11-month-old toddler was referred to our Pediatric Emergency Unit for a one-week history of rhinitis and two episodes of vomiting. His prior clinical history was unremarkable. On clinical examination an acute upper respiratory infection was diagnosed, with palpable reactive bilateral cervical lymph nodes. During clinical examination a swelling located on the left submandibular area was found and an enlargement of the left submandibular gland was suspected. The swelling was painless, soft, non-pulsatile and non-fluctuating without any signs of inflammation. Blood tests were normal, showing no increase of inflammatory markers nor immunological abnormalities. A soft-tissue ultrasound of the mass was performed, showing an enlargement of the left submandibular gland, reactive lymph nodes in the left cervical area and into the parotid parenchyma, and a 3.5 × 1.7 cm hypoechoic irregular lesion, with intralesional vascular signs and punctate and linear echogenic foci resulting in a “speckled” or “salt and pepper” pattern (Figure 4a).

The echo pattern of this mass was identical to that of the normal thymus located in the superior mediastinum, thus the diagnosis of ECT was suggested. Additionally, the ultrasound showed a portion of thymic tissue extending into deeper layers, a probable remnant of its migration during embryogenesis, aiding in its identification (Figure 4b).

**Figure 5:**
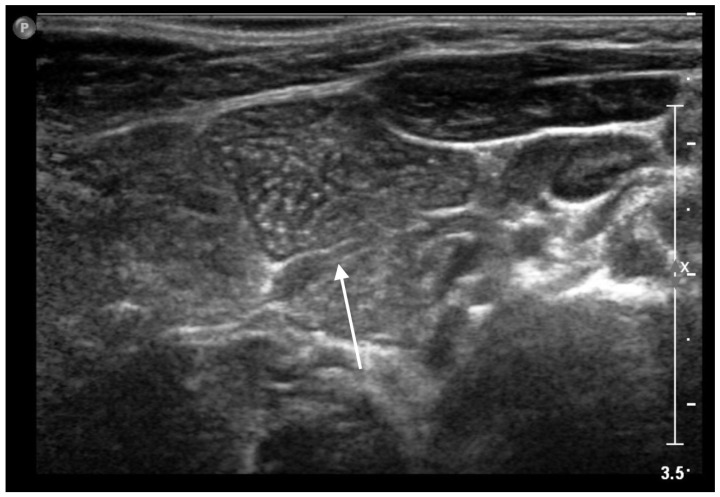
Follow-up ultrasound showing a slight reduction in size (2.6 × 1.7 cm) of the ectopic cervical thymus (*white arrow*).

The ultrasound examination was effective in making a prompt and confident diagnosis of an abnormal thymus location, without the need of further and potentially risky investigations. A radiological and clinical follow-up was started. At the 11-month follow-up, far from the acute upper respiratory infection, a slight reduction in size was observed, without any symptoms (Figure 5).

## 4. Discussion

This paper describes three pediatric patients with cervical mass due to abnormal thymus locations, highlighting the benignity of these congenital anomalies, particularly given the physiologic involution of the thymus that makes them transient.

The thymus is physiologically located at the level of the anterior mediastinum, behind the sternum, anterior to the aorta and jugular artery and inferior to the sternocleidomastoid muscle. Anomalies during embryogenesis or migration could be responsible for the occurrence of location thymus abnormalities, such as ECT, intrathyroidal ectopic thymus or the superior herniation of a normal mediastinal thymus.

The ECT is usually located along a line extending from the angle of the mandible to the manubrium sterni [5], although it can be found anywhere along the thyropharyngeal duct, which follows the embryonic descent of the thymus from the neck to the mediastinum. ECT appears as a palpable mass that can be tender, solid, cystic or mixed [6]. Differential diagnosis includes several conditions, such as fibromatosis colli, cervical lymphadenopathy, dermoid, epidermoid, hemangioma, neuroblastoma, ectopic thyroid and other metastatic lesions [7].

Most cases of ECT are diagnosed between 2 and 13 years of age, because the thymic parenchyma exhibits hyperplasia during the first decade of life or following infection or vaccination, making ECT more evident during early childhood, followed by a gradual regression due to physiologic involution of the thymus [8]. According to this, in our Case 3, ECT was diagnosed during an episode of acute upper respiratory infection. ECT is more common in male children and most commonly located on the left side of the neck [9].

Another rare and peculiar manifestation of thymus location abnormalities is its herniation into the neck from the anterior mediastinum. A transient swelling above the suprasternal notch appears when an increase in intrathoracic pressure occurs, such as during straining, crying or, as in our Case 1, coughing [10]. The dynamic ultrasound shows a normal mediastinal thymus that moves in the anterior part of the neck during the Valsalva maneuver [11]. Even though the exact mechanism is not known yet, Wong et al. [12] suggested that the connective tissue surrounding this organ could be exceptionally loose, leading to an upper migration of the thymus during conditions with an increased intrathoracic pressure. Because of its location and its manifestation as a cervical mass, thymus herniation can raise concern in parents and caregivers. However, its appearance only during Valsalva maneuver helps in the differential diagnosis, which includes thyroglossal duct cyst, branchiogenic cysts, cervical ectopic thymus, teratoma, cystic hygroma, thyroid tumor and others [13].

In most of cases, thymus location abnormalities are asymptomatic. When the child presents with symptoms suggesting a mass effect, such as stridor, dyspnea and dysphagia, clinicians should suspect a more threatening condition, needing further investigations.

The ultrasound has a pivotal role in the assessment of thymus location anomalies. In fact, the peculiarity of its echotexture is essential to detect thymic parenchyma in locations other than anterior mediastinum [1]. On ultrasound the thymus appears as a homogeneous, hypoechoic mass with multiple echogenic structures and internal echogenicity [14]. Commonly, the ultrasound appearance of thymus is described as stippled “starry sky” or “dot and dash” appearance, probably due to the hypererchoic fat against the background of the remaining hypoechoic lymphoid tissue [15]. In an ultrasound-pathology correlation study of intrathymic anatomy, the echogenic linear structures within the thymus were found to reflect the connective-tissue septa and the blood vessels in the septa, showing a high correlation between characteristic ultrasound features and the diagnosis of ectopic thymic tissue, then confirmed in more recent studies [14,16]. On Doppler sonography, blood flow was demonstrated in many of these echogenic lines and foci suggesting, as expected, the presence of blood vessels in the connective-tissue septa [14].

While normal mediastinal thymus could be susceptible to shape modification due to respiratory or cardiac contractions, ECT tends to maintain its own shape. Moreover, in ECT or intrathyroid thymus ultrasound can occasionally show a line of thymic tissue connecting it to the orthotopic thymus or simply a probable remnant of its migration during embryogenesis, aiding its identification.

Other imaging studies including computed tomography (CT) or MRI are often performed in pediatric patients with cervical masses, even though in selected cases an accurate ultrasound can be sufficient for the diagnosis. When the typical thymic “starry sky” appearance is detected on ultrasound, no signs or symptoms consistent with mass effect are reported and when the mass does not show a rapid dimensional growth, additional, and potentially risky procedures (i.e., MRI under general anesthesia) or surgical interventions, such as the mass excision to perform a histological examination, may be unnecessary. A strict clinical and radiological follow-up is recommended, to document the mass reduction, due to the physiologic involution of the thymus.

In the past, a fine needle aspiration biopsy (FNAB) or surgical excision were commonly performed to confirm the diagnosis [2,17]. In more recent years, given the benignity of these conditions, when the thymus is found in locations other than anterior mediastinum, a conservative management is gaining ground, due to the absence of complications and the spontaneous tendency to regression. Moreover, as a result of the development and the spreading of imaging techniques, the surgical approach gradually appeared to be unnecessary in most cases. Nowadays, surgery may be avoided when the common clinical and radiological features of ECT are found. FNAB or excisional surgery may be necessary when the diagnosis is uncertain, even after the MRI, to rule out malignancy [14].

Moreover, indications for excisional surgery may be the evidence or the clinical suspicion of a mass effect (suggested by imaging or symptoms such as dyspnea or dysphonia) and the rapid dimensional growth of the lesion. Since thymus is a soft, pliable organ, that changes its shape in response to respiratory and cardiac motion, the evidence of compression of adjacent structures should raise the suspect of hyperplasia or malignancy, requiring a surgical approach [18].

However, thymectomy may be responsible for immunodeficiencies because of a dysregulation in the T-cell lines. For this reason, a preoperative MRI is recommended in order to confirm the presence of at least minimal remnants of thymic tissue in the mediastinum. In addition, the possible severe complications of the surgical approach, such as the rupture of the innominate artery, recurrent laryngeal nerve injuries or damage of adjacent structures in the upper mediastinum, should be considered before surgery [19].

## 5. Conclusions

In conclusion, this case series focuses on abnormal locations of thymic tissue that can be an uncommon cause of neck masses in pediatric patients. Hence, these conditions, including ECT, intrathyroidal ectopic thymus and the superior herniation of a normal mediastinal thymus, should be considered in the differential diagnoses in children evaluated for a cervical mass. Given their benignity and the peculiar ultrasonographic appearance of thymic tissue, in selected cases our paper supports a conservative and minimal invasive approach in both diagnosis and management of these conditions. When the typical thymic “starry sky” appearance is detected on ultrasonography, no signs or symptoms consistent with mass effect are reported and when the mass does not show a rapid dimensional growth, ultrasound should be preferred for the diagnosis and follow-up of these patients over other imaging studies, which may require general anesthesia or cause radiation exposure.

We highlighted these benign uncommon conditions in Pediatrics to be promptly recognized by health care providers to avoid additional investigations, family concern and unnecessary treatments.

## Data Availability

Not applicable.
